# Consensus pan-genome assembly of the specialised wine bacterium *Oenococcus oeni*

**DOI:** 10.1186/s12864-016-2604-7

**Published:** 2016-04-27

**Authors:** Peter R. Sternes, Anthony R. Borneman

**Affiliations:** The Australian Wine Research Institute, PO Box 197, Glen Osmond, South Australia 5064 Australia

**Keywords:** Comparative genomics, Oenococcus, Industrial microbiology, Pan-genome, Assembly, Amino acid, Phosphotransferase, Competence, Ortholog

## Abstract

**Background:**

*Oenococcus oeni* is a lactic acid bacterium that is specialised for growth in the ecological niche of wine, where it is noted for its ability to perform the secondary, malolactic fermentation that is often required for many types of wine. Expanding the understanding of strain-dependent genetic variations in its small and streamlined genome is important for realising its full potential in industrial fermentation processes.

**Results:**

Whole genome comparison was performed on 191 strains of *O. oeni*; from this rich source of genomic information consensus pan-genome assemblies of the invariant (core) and variable (flexible) regions of this organism were established. Genetic variation in amino acid biosynthesis and sugar transport and utilisation was found to be common between strains. Furthermore, we characterised previously-unreported intra-specific genetic variations in the natural competence of this microbe.

**Conclusion:**

By assembling a consensus pan-genome from a large number of strains, this study provides a tool for researchers to readily compare protein-coding genes across strains and infer functional relationships between genes in conserved syntenic regions. This establishes a foundation for further genetic, and thus phenotypic, research of this industrially-important species.

**Electronic supplementary material:**

The online version of this article (doi:10.1186/s12864-016-2604-7) contains supplementary material, which is available to authorized users.

## Background

*Oenococcus oeni*, formerly *Leuconostoc oenos*, is a member of the lactic acid bacteria (LAB) and is noted for its ability to perform malolactic fermentation (MLF) in wine, a de-acidification reaction in which malic acid is decarboxylated to lactic acid [1]. MLF is particularly common in the production of red wines (although MLF is used in some white wines) where the decarboxylation reaction and associated by-products impart favourable sensory characteristics and decrease the likelihood of spoilage [2–4]. *O. oeni* is found on grapes or in the natural environment at very low levels, but can be commonly found in the hostile environment of wine, where it readily grows in low pH, presence of alcohol and scarcity of nutrients that inhibit the growth of other microbes [5–7].

In addition to spontaneously occurring MLF, purified strains of *O. oeni* are commonly added to wine as starter cultures to enable more reliable secondary fermentation [1–4]. Specific strains are selected for this purpose based on production of desirable flavour compounds and/or resilience to stresses such as acidity, ethanol, sulfites and phenolic compounds. Understanding the genotypic attributes of this species is important for identifying these industrially-relevant phenotypes.

Recent genomic sequencing efforts [8–17] have revealed that *O. oeni* has a compact genome of approximately 1.8 Mb which presumably has resulted from specialisation of this microbe in the relatively narrow ecological niche of wine [18, 19]. Despite this streamlined genome, previous comparative genomic studies of *O. oeni* have shown substantial inter-strain genomic variation, with up to 10 % variation in protein coding genes between strains, including those participating in sugar utilisation and transport, exopolysaccharide biosynthesis and amino-acid biosynthesis [10, 12].

In contrast to the historical use of a single strain genome as the *de facto* “reference” for any one species, the ongoing reduction in the cost of whole-genome sequencing now allows for large numbers of representatives from within the same species to be sequenced. Intra-specific comparison of the variation in coding potential of these strains has led to the conceptualisation of the pan-genome – the full complement of genes for a species [20, 21]. Very recently, the first bacterial consensus “pan-chromosome” of *Acinetobacter baumannii* was assembled independent of any pre-assigned genome reference and identified both invariant (core) and variable (flexible) regions within the chromosome [22]. This allowed for the order and orientation of core genes and flexible genomic regions to be established and led to the characterisation and comparison of clusters of functionally-related genes.

In this study, we have utilised this approach to assemble the first *O. oeni* pan-genome. In conjunction with 49 previously described genome sequences, we sequenced the genomes of a further 142 strains from commercial and environmental sources. By utilising this expanded set of strains, we have broadened the scope and scale of genomic comparisons and provided a genetic basis for phenotypic characterisations of this industrially-important microbe. Specifically, we report the pan-genome assembly and phylogenomic association of regions predicted to encode intra-specific variations in amino acid biosynthesis, sugar transport and utilisation and natural competence.

## Results and Discussion

### Genome sequencing of *O. oeni*

Genome sequences of 187 wine isolates and four cider isolates, predominantly originating from Australia and France, but also from Lebanon, USA, Switzerland and England were used in this study, 49 of which have been previously characterised [10, 11]. On average, the additional 142 genome sequences were each assembled from 450,000 Illumina sequencing reads (300 bp, paired-end library) into 390 contigs, forming a consensus sequence of 1,970,000 bp in size and with 2200 predicted protein-coding sequences.

### Genetic diversity of *O. oeni*

**Fig. 1:**
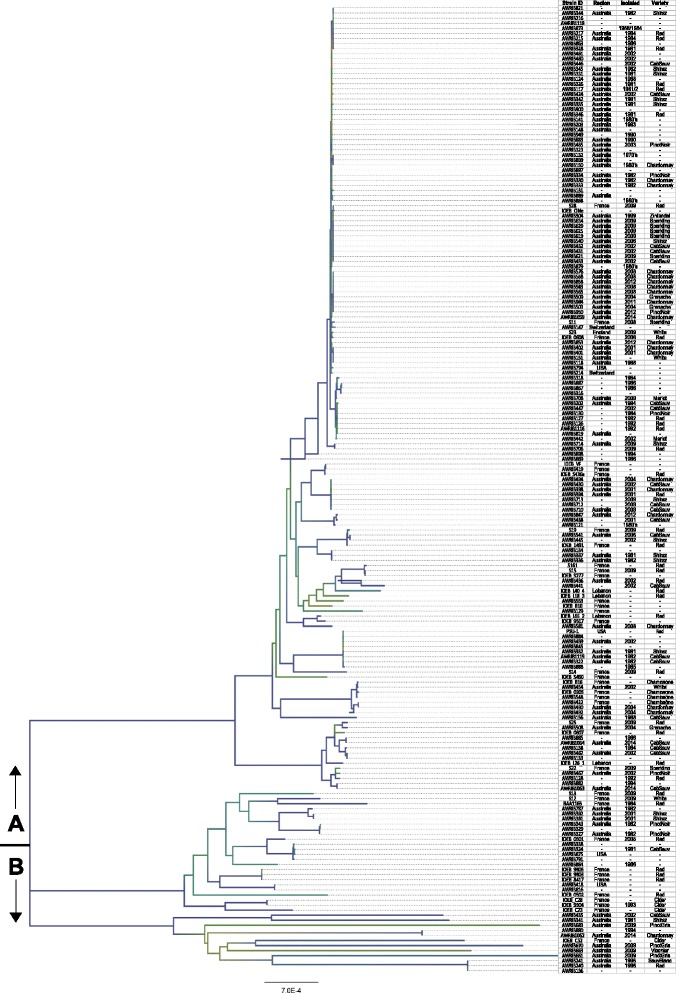
Neighbour-joining phylogeny based on whole-genome alignments of 191 *O. oeni* strains. The strain ID, region of isolation, date of isolation and the grape variety are as indicated. The tree was characterised into two broad genetic groups

Independent of coding-region predictions, the genetic relatedness of the various strains were deduced from the patterns of single-nucleotide polymorphisms (SNPs) from reference-based read mapping (Fig. 1). The resulting neighbour-joining dendrogram could be broadly split into two major genetic groups (A and B). Group A was in turn comprised of two subgroups; one genetically diverse and a second that contained a very large number of highly-related strains. Relative to Group A, Group B represents a highly-divergent clade comprised of genetically-distant strains. Consistent with previous reports [19], three out of four of the cider isolates cluster closely together in this group. However, while it has been suggested that this reflects domestication of *O. oeni* in a cider environment, the presence of numerous neighbouring wine-derived strains suggests that information from additional strains isolated from cider is required before any conclusions regarding the possibility of a cider-specific subset of *O. oeni* can be reached.

The variety, region and year of isolation did not appear to influence the clustering of strains into specific groups with the exception of the upper, closely-related group which is mostly comprised of Australian isolates (Fig. 1, Group A). Approximately 60 % of the known Australian isolates, but only 15 % of the known non-Australian isolates clustered into this genetic group. Strains isolated from France, Switzerland and the USA were also found in this closely-related group, making assertions about the ancestral geographic origin of these strains difficult. Unfortunately, very little is known regarding the stage of fermentation these strains were isolated from. Strains in this group may represent a robust variety that is capable of out-competing other strains during fermentation. Sampling late in the fermentation would therefore result in over-representation of this phylotype. Another possibility is that strains in this group are well suited to Australian winemaking conditions and the enrichment of Australian isolates in this genetic group is actually an accurate representation of the broader Australian population. As the concept of regional identity is very important in the valuation of wine and can be influenced by the bacterial strains performing MLF, further investigation whether Australian wines are typically dominated by this very closely-related subset of *O. oeni* population compared to other geographic regions represents a research direction worth consideration.

### The *O. oeni* pan-genome

**Fig. 2:**
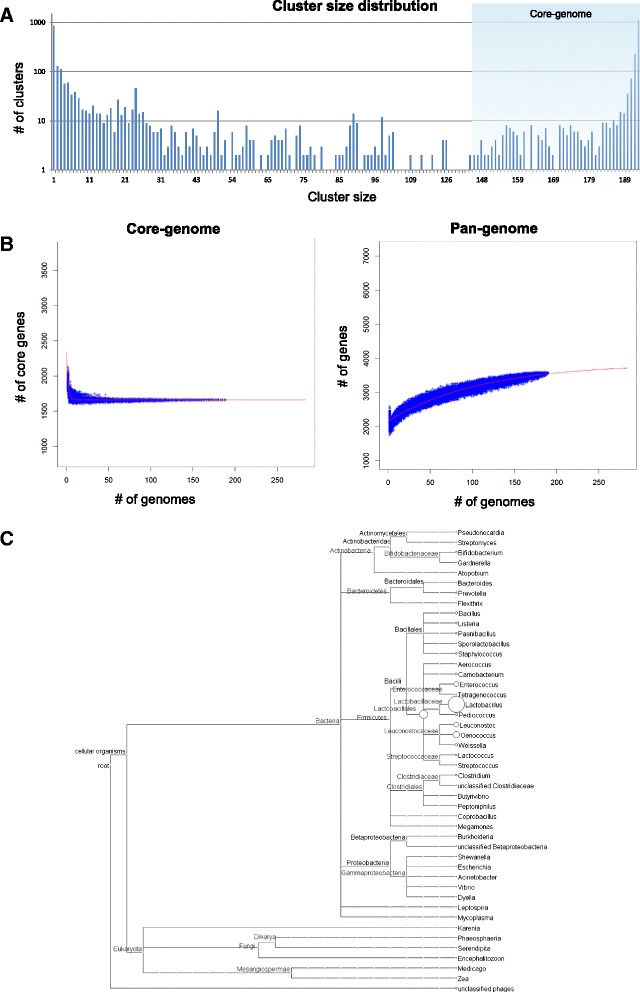
The core- and pan-genomes of *O. oeni*. **a**. Distribution of protein cluster sizes generated from the comparison of 191 genomes. **b**. Calculation of core- and pan-genome sizes including exponential law models to fit the medians. **c**. Distribution of BLAST best-hits by genus for clusters with no *O. oeni* match in the NCBI non-redundant dataset. The size of the circles represents the number of assigned hits

Previous comparative genomic studies of much smaller cohorts of *O. oeni* strains revealed substantial genomic diversity between some isolates [8–12, 23–25]. Despite these efforts, the full extent of the pan-genome remains unclear. The core- and pan-genome sizes of *O. oeni* were therefore determined for this large collection of strains using the pan-genome ortholog clustering tool, PanOCT [22, 26]. Unlike pure sequence-based clustering tools, PanOCT differentiates paralogous and non-paralogous ORFs using the conserved gene neighbourhood to separate duplicated gene families. Thus, singleton clusters can be formed from insertion sequence (IS) elements that are in novel contexts, even though the IS elements are identical [22]. In this context, there were 1661 core clusters (partial or complete ORF sequences in ≥75 % of the strains) and 1950 variable clusters assembled from the 191 strains (Fig. 2a).

In order to determine if the genetic diversity of *O. oeni* had been sufficiently sampled, medians and exponential law regressions were calculated from 500 randomly sampled combinations of 191 strains (Fig. 2b). The sequencing of approximately 50 strains was sufficient to estimate the final core-genome size of 1661 genes. The size of the pan-genome was predicted to continue to expand, albeit at a slowing rate, beyond the size calculated using 191 genomes (Fig. 2b), indicating that the *O. oeni* pan-genome is still “open”. In addition, to ensure that potential bias derived from oversampling of closely-related strains, such as those in Group A, did not confound the core- and pan-genome estimates, 60 of the very closely-related genomes in Group A were excluded from the analysis. The estimations of core- and pan-genome sizes were not substantially different when compared to analysis of the complete set of genomes, indicating a negligible bias in the original calculations (Additional file 1: Figure S1). Using 500 iterations of 100 randomly sampled genomes, the median core-genome sizes were 1659 and 1631, and median pan-genome sizes were 3150 and 3162 for the full set and partial set respectively.

Further genome sequencing is therefore expected to be required to characterise the entire spectrum of genetic diversity in *O. oeni*, however additional variation is likely to be rare. Whilst prioritising future sequencing towards strains from winemaking locales not currently represented in this study may accelerate the discovery of additional genetic diversity, it is interesting to note that phylogenomic analysis of a small number of *O. oeni* strains from Italy [13–15] and South America [16, 17], which were released after this analysis was initiated, indicate that these strains actually fall within the existing genomic clades and so may not provide a substantial amount of additional variation (Additional file 2: Figure S2).

The *O. oeni* genome has previously been described to contain regions likely to have been horizontally-acquired from members of the *Lactobacillales* [10]. To infer the evolutionary relationship of *O. oeni* on a larger complement of strains, BLAST best hits were attributed to each cluster. A total of 329 clusters (9 %) did not display *O. oeni* as a best match in the NCBI non-redundant dataset. As could be expected, all of these clusters were found within the variable (non-core) genome and indicate new ORFs that have previously not been identified in other annotated strains of *O. oeni*. These non-*O. oeni* clusters appear to originate from members of *Lactobacillales* family, particularly the genera *Lactobacillus*, *Oenococcus*, *Leuconostoc* and *Enterrococcus* (Fig. 2c). A comparatively small number of clusters appear to originate from outside *Lactobacillales*, including members of the *Bacillales* and *Bacteroidales* families, and the phyla *Actinobacteria*, *Bacteroidetes* and *Proteobacteria* (Fig. 2c).

### Pan-genome assembly

Substantial genomic variation in exopolysaccharide and amino acid biosynthesis, and sugar transport and utilisation have been reported previously for *O. oeni* [10]. Amongst bacteria, these variations are often due to the insertion of mobile elements or variable regions described as flexible genomic islands (fGIs), which usually contain highly conserved ORFs from bacteriophage [27–34]. Establishing a syntenic order of sequences was therefore critical for determining the orthology of genomic regions and to reflect important functional relationships between genes [35–37].

**Fig. 3:**
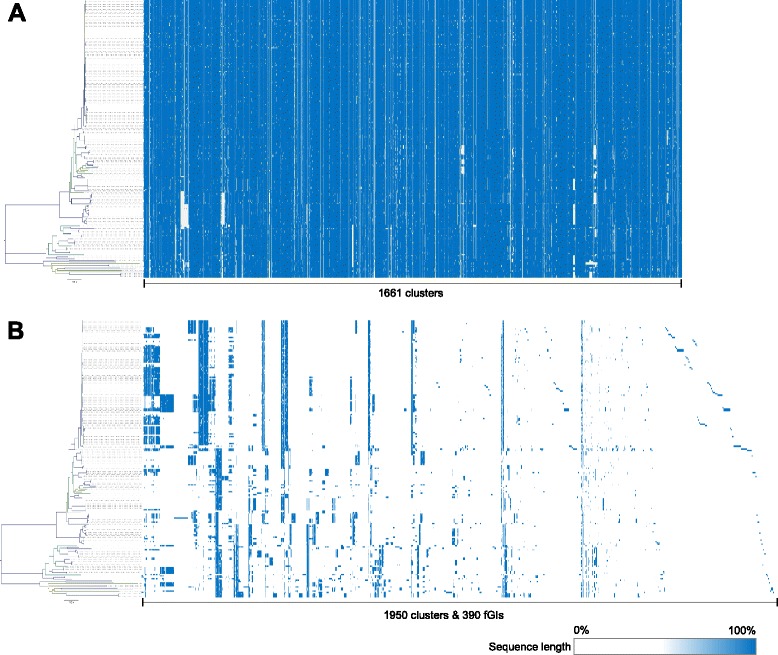
Visualisation of the core-genome and fGI assemblies. Full versions of the annotated assemblies are available in Additional file 3. **a**. Core-genome assembly of 1661 clusters. **b**. Concatenated fGI assemblies of 1950 clusters into 390 fGIs

To capture this information, a consensus core-genome and fGI assemblies were computed for the *O. oeni* pan-genome as described by Chan et al. [22] (Additional file 3). This methodology links clusters together based on the consensus of the layout of ORFs in individual *de novo* genome assemblies. In this study, the PSU-1 strain was used as a basal reference sequence to initially guide the arrangement of the clusters and this ultimately resulted in a core-genome assembly that closely resembles the arrangement of the PSU-1 genome (Fig. 3a). To help elucidate whether individual ORFs in a cluster are likely to be truncated, the lengths of each peptide sequence were calculated as a percentage of the longest peptide sequence in the cluster (Additional file 3).

Clusters at the beginning and end of the core-genome assembly (defined as a composition of clusters which contain sequences from at least 75 % of the strains) were linked, indicating assembly into a circular topology, as expected. The core-genome assembly revealed several large regions, up to 25 ORFs in size, which were completely absent in some clades of the genetic relatedness dendrogram (Fig. 3a, Additional file 3).

In addition to generating an assembled consensus core-genome, fGIs were also assembled. The fGIs were exclusively linear in topology and were located in specific clades of the relatedness dendrogram (Fig. 3b, Additional file 3). A total of 1950 clusters were assembled into 390 fGIs, the largest of which representing a bacteriophage insertion containing 52 ORFs. Interestingly, several fGIs were found to be unique to the closely related genetic group that consists mostly of Australian isolates.

### Amino acid biosynthesis

*O. oeni* has previously been reported to exhibit a variety of amino acid auxotrophies, with many strains showing intra-specific genomic differences [10, 38–44]. Early genomic analysis of the PSU-1 strain using the COG database [45] suggested the capacity for biosynthesis of eight amino acids: alanine, aspartate, asparagine, cysteine, glutamine, lysine, methionine and threonine [9]. In this expanded collection of strains and utilising KEGG, RAST and BLAST annotations, pathways leading to the biosynthesis of nine amino acids were observed in at least one strain (Table 1).

**Table 1:** Complete amino acid biosynthesis pathways in *O. oeni*

Amino acid	Pathway	KEGG ID
Glutamine	Glutamate to Glutamine	K01915
Glycine	Serine to Glycine	K00600
Serine	Glycine to Serine	K00600
Cysteine	Serine to Cysteine	M00021
Proline	Glutamate to Proline	M00015
Aspartate	Asparagine to Aspartate	K01424
	Lactate to Aspartate	K00016
		K01006
		K01595
		K14454
	Citrate to Aspartate	K01643
		K01644
		K01646
		K14454
	Malate to Aspartate	K00027
		K01006
		K01595
		K014454
Threonine	Aspartate to Threonine	M00018
Arginine	Glutamine to Arginine	K01955
		K01956
		K00611
		K01940
		K01755
Leucine	Valine to Leucine	M00432

**Fig. 4:**
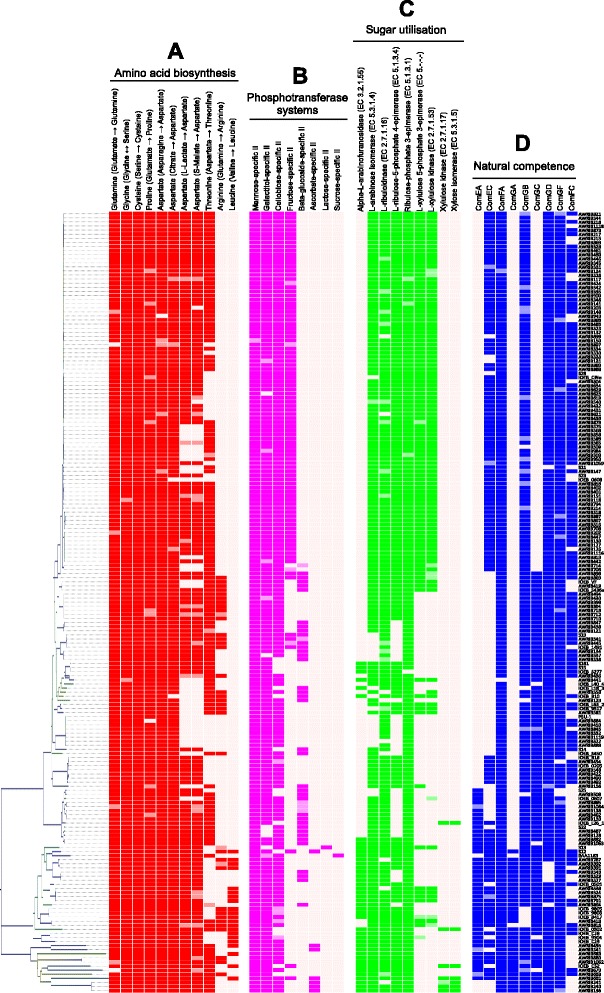
Intra-specific differences in amino acid biosynthesis, sugar transport and utilisation and natural competence. ORFs which contained a contig break are shaded in a lighter colour. **a**. Intra-specific differences in amino acid biosynthesis. Each pathway requires multiple enzymes, as described by their KEGG module numbers. **b**. Intra-specific differences in PTS components. Each sugar-specific system requires multiple subunits (typically IIA, IIB, IIC and occasionally IID). **c**. Intra-specific differences in the genes involved in five-carbon sugar utilisation, as described in Fig. 6. **d**. Intra-specific differences in the genes encoding natural competence proteins

**Fig. 5:**
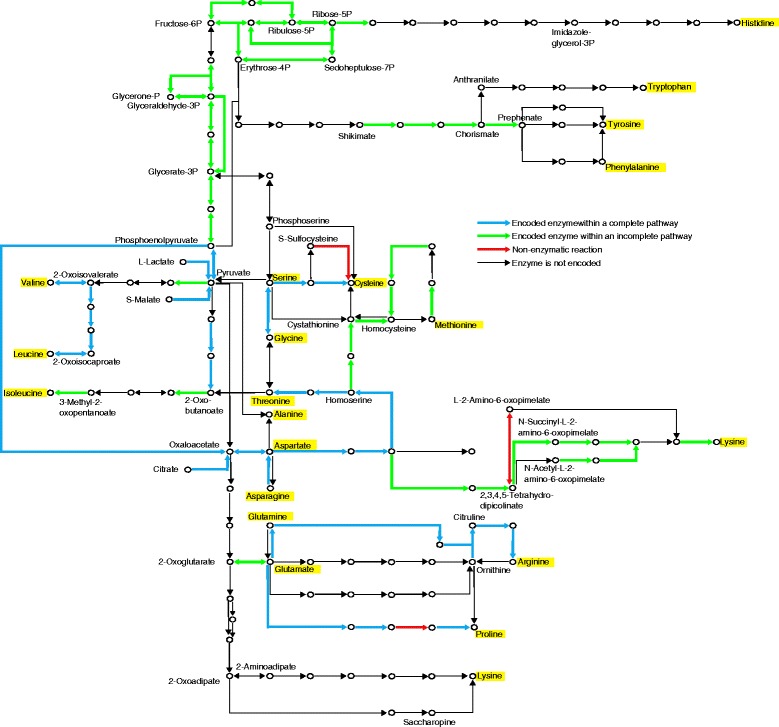
Overview of amino acid biosynthesis pathways in *O. oeni.* KEGG, RAST and BLAST annotations were used determine the presence of ORFs associated with amino acid biosynthesis across 191 strains. Pathways containing the full set of required genes, mostly between two amino acids (highlighted in yellow), are highlighted in blue and represented in Fig. 4a. ORFs forming incomplete pathways are highlighted in green. Pathways to make nine different amino acids were observed

The presence or absence of the complete sets of enzymes for each of these pathways in each strain was compiled and correlated with the genetic relatedness dendrogram (Fig. 4a) and highlighted in a pathway overview (Fig. 5). The complete pathways to synthesise glutamine, glycine, serine, cysteine, proline, aspartate and threonine were found to be conserved across the majority of strains. The ability to synthesise aspartate from lactic and malic acids was predicted to be disrupted in certain phylogenomic clades due to the presence of a frameshift mutation in pyruvate orthophosphate dikinase (EC 2.7.9.1), which is responsible for the conversion of pyruvate into phosphoenolpyruvate. Threonine biosynthesis deficiencies were also observed in specific clades. Loss of threonine biosynthesis capability exhibited intra-specific differences, as the deficient enzyme varied between strains and particularly in homoserine kinase (EC 2.7.1.39) where two different truncated versions of the peptide sequence were observed. The ability to synthesise leucine and arginine was predicted to exist in a small proportion of strains and was typically restricted to several small clades. Loss of a functional leucine biosynthesis pathway was attributed to mutations within 3-isopropylmalate dehydrogenase (EC 1.1.1.85) and isoproylmalate isomerase (EC 4.2.1.33). Similar to threonine biosynthesis, loss of the complete arginine biosynthesis pathway is attributed to several different mutations within genes from throughout the entire pathway. The ability to synthesise a tenth amino acid, alanine from aspartate, remains a possibility in three strains (AWRIB879, AWRIB708, AWRIB202) since the ORF encoding an aspartate 4-decarboxylase (EC 4.1.1.12) was found in fGIs excluded from the assembly because they contained less than three ORFs. In addition to these biosynthesis pathways, several incomplete pathways were also observed (Table 2, Fig. 5).

**Table 2:** Incomplete amino acid biosynthesis pathways in *O. oeni*

Amino acid	Pathway	KEGG ID
Asparagine	Aspartate to Asparagine	K01914
Glutamate	Pyruvate to Glutamate	K01958
		K01647
		K01681
		K00031
		K14454
	Histidine to Glutamate	M00045
Histidine	Phosphoribosyl to Histidine	M00026
Isoleucine	Threonine to Isoleucine	M00570
Lysine	Aspartate to Lysine	M00525
		M00527
		M00526
		M00016
Methionine	Aspartate to methionine	M00017
Phenylalanine	Chorismate to Phenylalanine	M00024
Tryptophan	Chorismate to Tryptophan	M00023
Tyrosine	Chorismate to Tyrosine	M00025
Valine	Pyruvate to Valine	M00019

Since amino acid concentrations are low in wine, amino acid biosynthesis capabilities are considered to be an important growth requirement. Early phenotypic studies predicted between five and thirteen amino acids to be essential for the growth of different strains of *O. oeni* [39–41]. Furthermore at least two organic acids, malic and citric acid, were involved in the biosynthesis of aspartate-derived amino acids [42]. A recent study which utilised a more sensitive methodology reported that two different *O. oeni* strains were auxotrophic for 13 and 16 amino acids, respectively [43]. Of the 16 essential amino acids found in one of these strains, only 8 were found to be essential in alternate strains from previous phenotypic studies, possibly reflecting substantial intra-specific variation. Of the 191 genomes analysed in this report, 11 to 15 amino acids were predicted to be unable to be converted from other amino acids or organic acids (Fig. 4a, Fig. 5).

*O. oeni* and other lactic acid bacteria are often described as having exacting nutritional requirements. Commercially used started cultures are often selected based on their resilience to wine stress conditions such as ethanol concentration, pH and temperature. Despite this, the rate of MLF can be substantially affected by nutrient availability [44], often resulting in sluggish or stuck fermentations. Comprehensive characterisation of amino acid auxotrophies can be useful for identifying essential nutritional requirements to help assess the suitability of wines or added nutrients for microbial growth and fermentability. Furthermore, it may also be used to assess the microbial stability of finished wines [43].

### Phosphotransferase enzyme systems in fGIs

The range of sugars that *O. oeni* is capable of utilising is strain dependent [46]. Previous studies have revealed intra-specific variation in the phosphotransferase system (PTS) enzyme II sugar transporters [25]. Similar to the characterisations of amino acid biosynthesis, variation in PTS enzyme II components (typically consisting of IIA, IIB, IIC and occasionally IID subunits) were analysed in this expanded set of strains (Fig. 4b). Four phosphotransferases, containing all of the required subunits, were conserved in the majority of strains: mannose-specific II, galactitol-specific II, cellobiose-specific II and beta-glucoside-specific II. Two phosphotransferases were observed to correspond to specific clades: the fructose-specific II and ascorbate-specific II. The full complement of subunits of the fructose-specific II transporter was conferred by the presence of an fGI encoding fructose-specific IIB and IIC components. This fGI was comparatively large with 29 ORFs encoding various cell wall related proteins (Additional file 4: Figure S3A) and generally corresponded to the Group A clade. For the ascorbate-specific II transporter, the majority of strains encoded the ascorbate-specific IIA and IIC subunits however only certain clade-specific strains encoded the ascorbate-specific IIB subunit.

In addition to these differences, two highly strain-specific phosphotransferases were observed: sucrose-specific II and lactose-specific II. The sucrose-specific IIA and IIBC subunits occurred in an fGI specific to the strain BAA-1163. Upon further investigation, the fGI encoding these subunits was predicted to also encode additional sucrose-related proteins including sucrose operon repressors and both a partial and complete sucrose-6-phosphate hydrolase. This group of ORFs would therefore be expected to allow for the perception, transport and metabolism of sucrose (Additional file 4: Figure S3D). The ORF encoding the lactose-specific IIA component was predicted to be present only in the S13 strain whereas the IIB and IIC components were commonly found in other strains. It is interesting to note that despite *O. oeni* existing in a relatively specific ecological niche, this bacterium retains diversity in the specific collection of PTS systems encoded in each strain.

### Five-carbon sugar utilisation pathways in fGIs

**Fig. 6:**
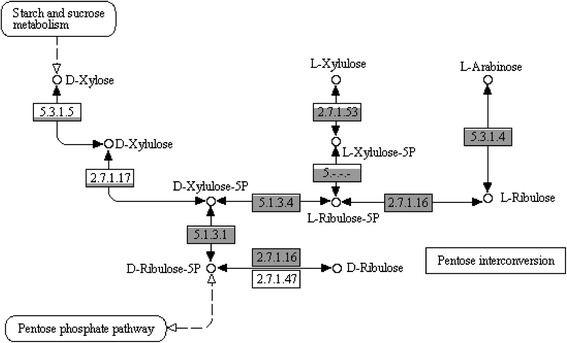
Variations in five-carbon sugar utilisation in *O. oeni*. The predicted pathways for the assimilation of xylose, arabinose and xylulose and the individual enzymatic steps with their corresponding EC numbers are indicated. The grey shading represents the number of strains out of 191 which contain that enzyme. The boxes are shaded relative to each other on a square-root scale. EC 2.7.1.16 was most common, present in 176 strains

It has been previously reported that *O. oeni* exhibits strain-dependent sugar utilisation phenotypes, particularly with the five-carbon sugars arabinose, xylulose and xylose and the metabolic pathways for arabinose and xylulose utilisation have previously been shown to be strain-specific [10, 46]. By comparing this larger set of strains, it was possible to define the extent of the arabinose and xylulose utilisation pathways (Fig. 4c and Fig. 6). L-arabinose utilisation is encoded by a set of three enzymes (L-arabinose isomerase EC 5.3.1.4, L-ribulokinase EC 2.7.1.16 and ribulose-phosphate 4-epimerase EC 5.1.3.1) (Fig. 6) which were present in the core-genome assembly, indicating that they were present in at least 75 % of the strains, however the enzyme required for the hydrolysis of the arabinose polymer arabinan (Alpha-N-arabinofuranosidase EC 3.2.1.55) was only found in a subset of strains predominantly found in Group B of the genetic relatedness dendrogram (Fig. 1 and Fig. 4c). Three enzymes responsible for L-xylulose utilisation (L-ribulose-5-phosphate 4-epimerase EC 5.1.3.4, L-xylulose 5-phosphate 3-epimerase EC 5.-.-.- and L-xylulokinase EC 2.7.1.53) (Fig. 6) were found to be encoded in adjacent positions within the same fGI (Additional file 4: Figure S3C) and generally appeared in a closely-related clade in Group A of Fig. 1.

In addition to these pathways, it was also possible to define an fGI that is predicted to encode for the ability to utilise D-xylose via the pentose phosphate pathway, the first time that this pathway has been described in *O. oeni*. This fGI is predicted to encode the two enzymes required to interconvert xylose to xylulose-5P (xylose isomerise EC 5.3.1.5 and xylulose kinase EC 2.7.1.17) (Fig. 6), in addition to a xylose transcriptional regulator and a D-xylose proton-symporter (XylT) and was generally confined to a single specific phylogenomic clade (Additional file 4: Figure S3B).

It is interesting to note that these two fGIs (Additional file 4: Figure S3B and C) correspond to different clades. Given that these genomic regions are not found in other clades, it is tempting to hypothesise that specialisation of *O. oeni* in an environment composed of residual five-carbon sugars like xylose and arabinose (i.e., in wine) has directed the acquisition of these regions in different instances throughout the course of evolution.

### Natural competence of *O. oeni*

**Fig. 7:**
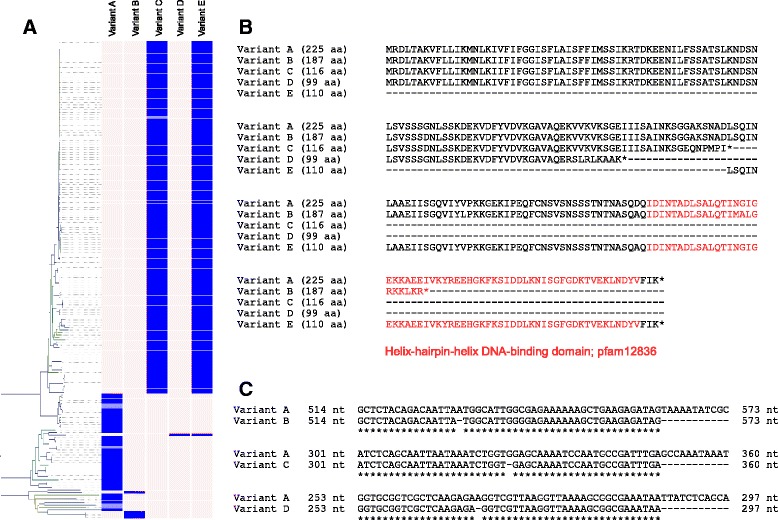
Intra-specific variation in the gene encoding the ComEA transmembrane DNA receptor. **a**. Five variants were found to map to specific branches of the genetic relatedness dendrogram. Strains which contained a contig break in the ORF encoding ComEA are shaded in light blue and assigned to the likely variant category. **b**. Alignment of predicted ComEA peptide sequences showing full-length (Variant A) and truncated (Variants B to E) versions. Variants B, C and D contained frameshift mutations resulting in prematurely-encoded stop codons which resulted in an additional ORF being predicted *in silico* (Variant E). The C-terminal DNA-binding motif is highlighted in red and is not encoded by Variants C and D. Variant B contains a premature stop within the DNA-binding domain and still corresponds with genetically-distant strains. Variant D represents a frameshift mutation unique to the BAA-1163 strain. **c**. Nucleotide sequence alignment highlighting single nucleotide deletions causing frameshift mutations and truncation of the ComEA peptide sequence

Many bacteria are naturally competent and able to actively transport environmental DNA fragments across their cell envelope and into their cytoplasm [47–52]. Competence represents an important mechanism to allow for horizontal gene transfer as well as providing access to nutrients. Uptake of extra-cellular DNA in Gram-positive bacteria, such as *O. oeni*, requires a suite of proteins which include DNA receptors (ComEA), transmembrane pores (ComEC), transformation pili (ComGC), ATP-dependent translocases (ComFA) and additional proteins encoded by the ComG operon. Substantial intra-specific diversity with *O. oeni* was observed for these natural competence proteins (Fig. 4d). Interestingly, the highly diverse clade (Group B in Fig. 1) retains full-length peptide sequences for proteins that appear truncated elsewhere on the tree. For example, strains in Group B mostly retained full-length versions of ComEA whereas other strains contained one of three different frameshift mutations in the gene encoding ComEA, all resulting in prematurely-encoded stop codons (Fig. 7). ComEA is a bitopic membrane protein often described as being obligatory for natural genetic transformations. ComEA consists of a transmembrane N-terminal domain and a C-terminal domain outside the cytoplasm membrane [53, 54]. The C-terminal domain contains a helix-hairpin-helix DNA-binding motif which is the structural basis for non-sequence-specific recognition of DNA [55]. Two of the three frameshift mutations preclude the entire DNA-binding motif from being encoded and this is anticipated to have an adverse effect on the ability of *O. oeni* to bind DNA from the extracellular environment. It is unknown whether the predicted ORF downstream of a premature stop is transcribed *in vivo* (Variant E, Fig. 7), however the loss of a large N-terminal end would presumably affect the functionality of this protein.

Conceivably, retention of the functional versions of ComEA and other competence proteins has allowed for a protracted evolutionary divergence of Group B, as evidenced by the higher inter-strain branch lengths in the phylogeny (Fig. 1). With the exception of ComGC, all the genes encoding these proteins were found in the core-genome assembly. Presence of the truncated versions of these proteins in the core-genome may indicate that most modern-day *O. oeni* strains share a naturally competent ancestor but have lost this competence by processes such as genome decay. Since *O. oeni* has become specialised to a relatively stable, abundant, simplified and less competitive ecological niche, the ability for it to adapt to environmental conditions by up taking extra-cellular DNA is presumably no longer essential for survival and may actually serve to disrupt its already specialised genome. To this day, the ability to reproducibly transform *O. oeni* for research purposes remains a considerable challenge. Given the intra-specific variations in their DNA uptake machinery, careful selection of strains which may be more amenable to transformation provides a sensible avenue for researchers to explore.

## Conclusions

Like other industrial species, phenotypic variation in *O. oeni* will have direct economic consequences through impacts on product quality and production efficiencies. This study has conducted the largest pan-genome analysis of *O. oeni* to date and expanded upon previous comparative genomic approaches by providing a consensus pan-genome assembly. The pan-genome assembly provides a powerful tool for researchers to compare protein-coding genes across a large number of strains with the added benefit of being able to infer likely functional relationships between genes in conserved syntenic regions. The applicability of the pan-genome assembly was demonstrated in this study by substantially expanding upon previous observations of intra-specific variation in amino acid biosynthesis and sugar transport and utilisation as well as characterising previously unreported variability in natural competence. Compilation of this vast amount of genomic information can be used to inform research on the industrial implications by allowing for identification of strains with combinations of desirable genetic, and therefore phenotypic, characteristics.

## Methods

### Strains and growth conditions

Strains used in this study are listed in Additional file 5. Strains were selected to represent a cross-section of commonly used commercial strains, in addition to Australian environmental isolates present in the AWRI culture collection. The strains were prepared by growing each strain in MRS (Amyl Media, Australia) supplemented with 20 % apple juice [56] for between six and ten days at 27 °C. DNA was prepared by phenol chloroform extraction as previously described [27].

### Genome sequencing and assembly

Genome sequencing was performed at the Ramaciotti Centre for Gene Function Analysis (University of New South Wales, NSW, Australia) using the Illumina MiSeq platform and 2 × 300 bp paired-end sequencing reads with a target depth of 60x coverage. For each strain, reads were assembled using MIRA (v 4.0rc5) [57] and potential coding regions were predicted using GLIMMER v3.0.2 [58]. These Whole Genome Shotgun projects have been deposited at DDBJ/EMBL/GenBank under the BioProject accession PRJNA304199.

Phylogenies were produced by aligning raw reads to the PSU-1 reference sequence [8, 9] using Novoalign v 3.02.12 (http://www.novocraft.com). Single nucleotide polymorphisms (SNPs) were called using Varscan v 2.3.8 [59] and were used to create strain-specific pseudo-genome sequences. Aligned pseudo-genomes were used as input for neighbour-joining dendrogram construction using Seaview4 v 4.4.2 [60].

### Pan-genome annotation and assembly

Clusters of orthologous proteins were generated by PanOct v 3.23 [22, 26] using default parameters. The resulting centroid sequences were annotated using BLAST [61], KAAS via the KEGG website [62] and RAST [63]. Consensus core and fGI assemblies of the pan-genome were calculated using the script, *gene_order.pl*, and the *75_core_adajacency_vector.txt* output from PanOCT. Spurious fGIs potentially due to random IS elements or bad gene calls defined as fGIs containing less than three ORFs were removed and compiled in Additional file 3. To eliminate redundancy, the core-genome centroids present at both ends of the fGI assemblies were trimmed. The core and fGI assemblies consisting of annotated centroids were then compiled into a spreadsheet (Additional file 3). The percentage length of each peptide relative to the longest peptide in the corresponding centroid was calculated and used to generate a heat map for the compiled assemblies. For intra-specific comparisons, such as summarised in Fig. 4, a functional version of an ORF was defined as an ORF length being >90 % of the length commonly represented for *O. oeni* in the NCBI non-redundant database.

The exponential law regressions to calculate the core- and pan-genome sizes were calculated from the output of PanOCT using the *compute_pangenome.R* and *plot_pangenome.R* scripts and randomly sampling without replacement 500 combinations of genomes.

## Availability of data and material

Sequencing reads and *de novo* genome assemblies are available under BioProject accession PRJNA304199. The pan-genome assembly and supporting information are available in supplementary files. All strains sequenced in this study are available through the Australian Wine Research Institute Culture Collection.

## Additional files

Additional file 1: Figure S1. Calculation of core- and pan-genome sizes including exponential law models to fit the medians. These calculations check for bias when a high number of closely-related strains are included in the core- and pan-genome size calculations. 60 closely-related genomes from Group A in Fig. 1 were excluded from the calculation to check for bias in Fig. 2b. (PDF 70 kb) Additional file 2: Figure S2. Updated neighbour-joining phylogeny to include recently released Italian and South American *O. oeni* strains. Neighbour-joining phylogeny based on whole-genome alignments of 191 *O. oeni* strains used for the pan-genome construction in addition to 10 strains from Italy (OM27, OM22, OT25, OT3, OT4, OT5), Argentina (XL2) and Chile (139, 399, 565) for which whole-genome data is now available. Phylogenomic clades containing the additional strains are highlighted in red. (PDF 10 kb) Additional file 3: Core-genome and fGI assemblies of ortholog clusters. A spreadsheet containing annotated and assembled ortholog clusters and their occurrence throughout all the strains analysed. The spreadsheet also contains a sheet including all the ortholog clusters filtered from the analysis. (XLSX 18748 kb) Additional file 4: Figure S3. fGIs conferring intra-specific differences in PTS enzymes and sugar utilisation. Graphical representation of four annotated fGIs and their phylogenomic relationship. A. A large fGI containing 29 genes, two of which encode fructose-specific IIB and IIC PTS components, completing the full suite of fructose-specific II components (IIA, IIB and IIC) in those strains. B. An fGI containing two enzymes, xylulose kinase EC 2.7.1.17 and xylose isomerise EC 5.3.1.5, and potentially related genes which is predicted to confer the ability to interconvert xylose to xylulose-5P. C. An fGI containing three enzymes, L-ribulose-5-phosphate 4-epimerase EC 5.1.3.4, L-xylulose 5-phosphate 3-epimerase EC 5.-,-,- and L-xylulokinase EC 2.7.1.53, and potentially related genes which is predicted to confer the ability to interconvert L-xylulose to D-xylulose-5P. D. A strain-specific fGI containing sucrose-related genes which encode sucrose-specific IIA and IIBC PTS subunits, sucrose operon repressors and partial and complete sucrose-6-phosphate hydrolases, potentially conferring the ability to perceive, transport and utilise sucrose. (PDF 1476 kb) Additional file 5: List of strains used in this study. Contains a list of strains and other relevant information. (XLSX 18 kb)

## Abbreviations

fGI: flexible genomic island
IS: insertion sequence
PTS: phosphotransferase system
SNP: single-nucleotide polymorphisms

## References

[1] Henick-Kling T, Fleet GH (1993). Malolactic fermentation. Wine microbiology and biotechnology.

[2] Bartowsky EJ, Henschke PA (2004). The “buttery” attribute of wine–diacetyl–desirability, spoilage and beyond. Int J Food Microbiol.

[3] Lonvaud-Funel A (1999). Lactic acid bacteria in the quality improvement and depreciation of wine. Antonie Van Leeuwenhoek.

[4] Swiegers JH, Bartowsky EJ, Henschke PA, Pretorius IS (2005). Yeast and bacterial modulation of wine aroma and flavour. Aust J Grape Wine Res.

[5] Bae S, Fleet GH, Heard GM (2006). Lactic acid bacteria associated with wine grapes from several Australian vineyards. J Appl Microbiol.

[6] Renouf V, Claisse O, Lonvaud-Funel A (2005). Understanding the microbial ecosystem on the grape berry surface through numeration and identification of yeast and bacteria. Aust J Grape Wine Res.

[7] Renouf V, Claisse O, Lonvaud-Funel A (2007). Inventory and monitoring of wine microbial consortia. Appl Microbiol Biotechnol.

[8] Makarova K, Slesarev A, Wolf Y, Sorokin A, Mirkin B, Koonin E, Pavlov A, Pavlova N, Karamychev V, Polouchine N, Shakhova V, Grigoriev I, Lou Y, Rohksar D, Lucas S, Huang K, Goodstein DM, Hawkins T, Plengvidhya V, Welker D, Hughes J, Goh Y, Benson A, Baldwin K, Lee J-H, Díaz-Muñiz I, Dosti B, Smeianov V, Wechter W, Barabote R, Lorca G, Altermann E, Barrangou R, Ganesan B, Xie Y, Rawsthorne H, Tamir D, Parker C, Breidt F, Broadbent J, Hutkins R, O’Sullivan D, Steele J, Unlu G, Saier M, Klaenhammer T, Richardson P, Kozyavkin S, Weimer B, Mills D (2006). Comparative genomics of the lactic acid bacteria. Proc Natl Acad Sci U S A.

[9] Mills D, Rawsthorne H, Parker C, Tamir D, Makarova K (2005). Genomic analysis of PSU-1 and its relevance to winemaking. FEMS Microbiol Rev.

[10] Borneman AR, McCarthy JM, Chambers PJ, Bartowsky EJ (2012). Comparative analysis of the Oenococcus oeni pan genome reveals genetic diversity in industrially-relevant pathways. BMC Genomics.

[11] Dimopoulou M, Vuillemin M, Campbell-Sills H, Lucas PM, Ballestra P, Miot-Sertier C, Favier M, Coulon J, Moine V, Doco T, Roques M, Williams P, Petrel M, Gontier E, Moulis C, Remaud-Simeon M, Dols-Lafargue M (2014). Exopolysaccharide (EPS) Synthesis by *Oenococcus oeni*: From Genes to Phenotypes. PLoS ONE.

[12] Borneman AR, Bartowsky EJ, McCarthy J, Chambers PJ (2010). Genotypic diversity in Oenococcus oeni by high-density microarray comparative genome hybridization and whole genome sequencing. Appl Microbiol Biotechnol.

[13] Lamontanara A, Orrù L, Cattivelli L, Russo P, Spano G, Capozzi V (2014). Genome Sequence of Oenococcus oeni OM27, the first fully assembled genome of a strain isolated from an Italian wine. Genome Announc.

[14] Capozzi V, Russo P, Beneduce L, Weidmann S, Grieco F, Guzzo J, Spano G (2010). Technological properties of Oenococcus oeni strains isolated from typical southern Italian wines. Lett Appl Microbiol.

[15] Capozzi V, Russo P, Lamontanara A, Orrù L, Cattivelli L, Spano G (2014). Genome sequences of five Oenococcus oeni strains isolated from Nero di Troia Wine in Apulia, Southern Italy. Genome Announc.

[16] Mendoza LM, Saavedra L, Raya RR (2015). Draft genome sequence of Oenococcus oeni strain X2L (CRL1947), isolated from red wine of northwest Argentina. Genome Announc.

[17] Jara C, Romero J (2015). Genome sequences of three Oenococcus oeni strains isolated from Maipo Valley, Chile. Genome Announc.

[18] Gibbons JG, Rinker DC (2015). The genomics of microbial domestication in the fermented food environment. Curr Opin Genet Dev.

[19] Campbell-Sills H, El Khoury M, Favier M, Romano A, Biasioli F, Spano G, Mariette El Khoury, Marion Favier, Andrea Romano, Franco Biasioli, Giuseppe Spano, David J. Sherman, Olivier Bouchez, Emmanuel Coton, Monika Coton, Sanae Okada, Naoto Tanaka, Marguerite Dols-Lafargue and Patrick M. Lucas. Phylogenomic Analysis of Oenococcus oeni Reveals Specific Domestication of Strains to Cider and Wines. Genome Biol Evol. 2015;7(6):1506–18. 10.1093/gbe/evv084 PMC4494047 25977455

[20] Tettelin H, Masignani V, Cieslewicz MJ, Donati C, Medini D, Ward NL, Angiuoli SV, Crabtree J, Jones AL, Durkin AS, Deboy RT, Davidsen TM, Mora M, Scarselli M, Margarit y Ros I, Peterson JD, Hauser CR, Sundaram JP, Nelson WC, Madupu R, Brinkac LM, Dodson RJ, Rosovitz MJ, Sullivan SA, Daugherty SC, Haft DH, Selengut J, Gwinn ML, Zhou L, Zafar N, Khouri H, Radune D, Dimitrov G, Watkins K, O'Connor KJ, Smith S, Utterback TR, White O, Rubens CE, Grandi G, Madoff LC, Kasper DL, Telford JL, Wessels MR, Rappuoli R, Fraser CM (2005). Genome analysis of multiple pathogenic isolates of Streptococcus agalactiae: implications for the microbial “pan-genome*”*. Proc Natl Acad Sci U S A.

[21] Tettelin H, Riley D, Cattuto C, Medini D (2008). Comparative genomics: the bacterial pan-genome. Curr Opin Microbiol.

[22] Chan AP, Sutton G, DePew J, Krishnakumar R, Choi Y, Huang X-Z, Harkins DM, Kim M, Lesho EP, Nikolich MP, Fouts DE. A novel method of consensus pan-chromosome assembly and large-scale comparative analysis reveal the highly flexible pan-genome of Acinetobacter baumannii. Genome Biol. 2015;16:143–70. 10.1186/s13059-015-0701-6 PMC4507327 26195261

[23] Bridier J, Claisse O, Coton M, Coton E, Lonvaud-Funel A (2010). Evidence of distinct populations and specific subpopulations within the species Oenococcus oeni. Appl Environ Microbiol.

[24] Bon E, Delaherche A, Bilhère E, De Daruvar A, Lonvaud-Funel A, Le Marrec C (2009). Oenococcus oeni genome plasticity is associated with fitness. Appl Environ Microbiol.

[25] Barabote RD, Saier MH (2005). Comparative genomic analyses of the bacterial phosphotransferase system. Microbiol Mol Biol Rev.

[26] Fouts DE, Brinkac L, Beck E, Inman J, Sutton G (2012). PanOCT: automated clustering of orthologs using conserved gene neighborhood for Pan-genomic analysis of bacterial strains and closely related species. Nucleic Acids Res.

[27] Zavaleta AI, Martínez-Murcia AJ, Rodríguez-Valera F (1997). Intraspecific genetic diversity of Oenococcus oeni as derived from DNA fingerprinting and sequence analyses. Appl Environ Microbiol.

[28] Fouts DE, Mongodin EF, Mandrell RE, Miller WG, Rasko DA, Ravel J, Brinkac LM, DeBoy RT, Parker CT, Daugherty SC, Dodson RJ, Durkin AS, Madupu R, Sullivan SA, Shetty JU, Ayodeji MA, Shvartsbeyn A, Schatz MC, Badger JH, Fraser CM, Nelson KE (2005). Major structural differences and novel potential virulence mechanisms from the genomes of multiple Campylobacter species. PLoS Biol.

[29] Gill SR, Fouts DE, Archer GL, Mongodin EF, Deboy RT, Ravel J, Paulsen IT, Kolonay JF, Brinkac L, Beanan M, Dodson RJ, Daugherty SC, Madupu R, Angiuoli SV, Durkin AS, Haft DH, Vamathevan J, Khouri H, Utterback T, Lee C, Dimitrov G, Jiang L, Qin H, Weidman J, Tran K, Kang K, Hance IR, Nelson KE, Fraser CM (2005). Insights on evolution of virulence and resistance from the complete genome analysis of an early methicillin-resistant Staphylococcus aureus strain and a biofilm-producing methicillin-resistant Staphylococcus epidermidis strain. J Bacteriol.

[30] Nelson KE, Fouts DE, Mongodin EF, Ravel J, DeBoy RT, Kolonay JF, Rasko DA, Angiuoli SV, Gill SR, Paulsen IT, Peterson J, White O, Nelson WC, Nierman W, Beanan MJ, Brinkac LM, Daugherty SC, Dodson RJ, Durkin AS, Madupu R, Haft DH, Selengut J, Van Aken S, Khouri H, Fedorova N, Forberger H, Tran B, Kathariou S, Wonderling LD, Uhlich GA, Bayles DO, Luchansky JB, Fraser CM (2004). Whole genome comparisons of serotype 4b and 1/2a strains of the food-borne pathogen Listeria monocytogenes reveal new insights into the core genome components of this species. Nucleic Acids Res.

[31] Chen Y, Stine OC, Badger JH, Gil AI, Nair GB, Nishibuchi M, Fouts DE (2011). Comparative genomic analysis of Vibrio parahaemolyticus: serotype conversion and virulence. BMC Genomics.

[32] Aydanian A, Tang L, Morris JG, Johnson JA, Stine OC (2011). Genetic diversity of O-antigen biosynthesis regions in Vibrio cholerae. Appl Environ Microbiol.

[33] Jacobsen A, Hendriksen RS, Aaresturp FM, Ussery DW, Friis C (2011). The Salmonella enterica pan-genome. Microb Ecol.

[34] Rodriguez-Valera F, Ussery DW (2012). Is the pan-genome also a pan-seletome?. F1000 Res.

[35] Moreno-Hagelsieb G, Treviño V, Pérez-Rueda E, Smith TF, Collado-Vides J (2001). Transcription unit conservation in the three domains of life: a perspective form *Escherichia coli*. Trends Genet.

[36] Dandekar T, Snel B, Huynen M, Bork P (1998). Conservation of gene order: a fingerprint of proteins that physically interact. Trends Biochem Sci.

[37] Overbeek R, Fonstein M, D'Souza M, Pusch GD, Maltsev N (1999). The use of gene clusters to infer functional coupling. Proc Natl Acad Sci U S A.

[38] Garvie EI (1967). Leuconostoc oenos sp.nov. J Gen Microbiol.

[39] Fourcassie P, Makaga-Kabinda-Massard E, Belarbi A, Maujean A (1992). Growth, D-glucose utilization and malolactic fermentation by *Leuconostoc oenos* strains in 18 media deficient in one amino acid. J Appl Bacteriol.

[40] Peynaud E, Lafon-Lafourcade S, Domercq S (1965). Besoins nutritionnels de 64 souches de bactéries lactiques isolées de vins. Bulletin de l'OIV.

[41] Remize F, Gaudin A, Kong Y, Guzzo J, Alexandre H, Krieger SA, Guilloux-Benatier M (2006). *Oenococcus oeni* preference for peptides: qualitative and quantitative analysis of nitrogen assimilation. Arch Microbiol.

[42] Saguir FM, de Nadra M (2002). Effect of L-malic and citric acids metabolism on the essential amino acid requirements for *Oenococcus oeni* growth. J Appl Microbiol.

[43] Terrade N, Mira de Orduña R (2009). Determination of the essential nutrient requirements of wine-related bacteria from the genera Oenococcus and Lactobacillus. Int J Food Microbiol.

[44] Gockowak H, Henschke P (2003). Interaction of pH, ethanol concentration and wine matrix on induction of malolactic fermentation with commercial ‘direct inoculation’ starter cultures. Aust J Grape Wine R.

[45] Tatusov RL, Galperin MY, Natale DA, Koonin EV (2000). The COG database: a tool for genome-scale analysis of protein functions and evolution. Nuc Acid Res.

[46] Dicks LM, Halzapfel WH, Vos P, Garrity G, Jones D, Krieg NR, Ludwig W, Rainey FA, Schleifer KH, Whitman WB (2009). Genus II. Oenococcus. *Bergey’s manual of systematic bacteriology*. Second edition.

[47] Lorenz MG, Wackernagel W (1994). Bacterial gene transfer by natural genetic transformation in the environment. Microbiol Rev.

[48] Chen I, Christie PJ, Dubnau D (2005). The ins and outs of DNA transfer in bacteria. Science.

[49] Krüger NJ, Stingl K (2011). Two steps away from novelty—principles of bacterial DNA uptake. Mol Microbiol.

[50] Chen I, Dubnau D (2004). DNA uptake during bacterial transformation. Nat Rev Microbiol.

[51] Seitz P, Blokesch M (2013). Cues and regulatory pathways involved in natural competence and transformation in pathogenic and environmental Gram-negative bacteria. FEMS Microbiol Rev.

[52] Johnsborg O, Eldholm V, Håvarstein LS (2007). Natural genetic transformation: prevalence, mechanisms and function. Res Microbiol.

[53] Provvedi R, Dubnau D (1999). ComEA is a DNA receptor for transformation of competent Bacillus subtilise. Mol Microbiol.

[54] Seitz P, Modarres HP, Borgeaud S, Bulushev RD, Steinbock LJ, Radenovic A, Dal Peraro M, Blokesch M (2014). ComEA Is Essential for the Transfer of External DNA into the Periplasm in Naturally Transformable Vibrio cholerae Cells. PLoS Genet.

[55] Doherty AJ, Serpell LC, Ponting CP (1996). The Helix-Hairpin-Helix DNA-Binding Motif: A Structural Basis for Non-Sequence-Specific Recognition of DNA. Nucleic Acids Res.

[56] Kelly WJ, Asmundson RV, Hopcroft DH (1989). Growth of Leuconostoc oenos under anaerobic conditions. Am J Enol Vitic.

[57] Chevreux B, Pfisterer T, Drescher B, Driesel AJ, Müller WEG, Wetter T, Suhai S (2004). Using the miraEST Assembler for Reliable and Automated mRNA Transcript Assembly and SNP Detection in Sequenced ESTs. Genome Res.

[58] Delcher AL, Bratke KA, Powers EC, Salzberg SL (2007). Identifying bacterial genes and endosymbiont DNA with Glimmer. Bioinformatics.

[59] Koboldt DC, Chen K, Wylie T, Larson DE, McLellan MD, Mardis ER, Weinstock GM, Wilson RK, Ding L (2009). VarScan: variant detection in massively parallel sequencing of individual and pooled samples. Bioinformatics.

[60] Gouy M, Guindon S, Gascuel O (2010). SeaView Version 4: A Multiplatform Graphical User Interface for Sequence Alignment and Phylogenetic Tree Building. Mol Biol Evol.

[61] Altschul SF, Madden TL, Schäffer AA, Zhang J, Zhang Z, Miller W, Lipman DJ (1997). Gapped BLAST and PSI-BLAST: a new generation of protein database search programs. Nucleic Acids Res.

[62] Moriya Y, Itoh M, Okuda S, Yoshizawa AC, Kanehisa M (2007). KAAS: an automatic genome annotation and pathway reconstruction server. Nucleic Acids Res.

[63] Overbeek R, Olson R, Pusch GD, Olsen GJ, Davis JJ, Disz T, Edwards RA, Gerdes S, Parrello B, Shukla M, Vonstein V, Wattam AR, Xia F, Stevens R (2014). The SEED and the Rapid Annotation of microbial genomes using Subsystems Technology (RAST). Nucleic Acids Res.

