# Erratum to: ‘Consensus pan-genome assembly of the specialised wine bacterium *Oenococcus oeni*’

**DOI:** 10.1186/s12864-016-2811-2

**Published:** 2016-10-20

**Authors:** Peter R. Sternes, Anthony R. Borneman

**Affiliations:** The Australian Wine Research Institute, PO Box 197, Glen Osmond, South Australia 5064 Australia

Unfortunately, the version of this article as originally published [1] contained an error. The quality of Figs. 1, 2, 3, 4, 5, 6 and 7 were affected due to an error in sampling the original images and an incorrect version of the article being used during the production process. For the sake of clarity, the legible Figs. 1, 2, 3, 4, 5, 6 and 7 have been included below.

**Fig. 1:**
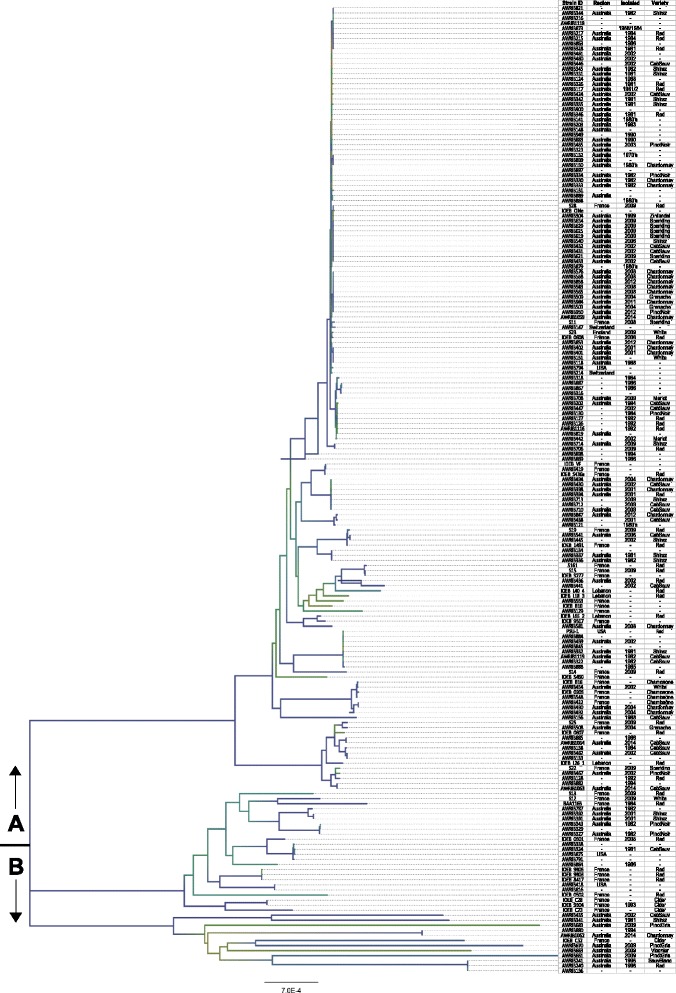
Neighbour-joining phylogeny based on whole-genome alignments of 191 *O. oeni* strains. The strain ID, region of isolation, date of isolation and the grape variety are as indicated. The tree was characterised into two broad genetic groups

**Fig. 2:**
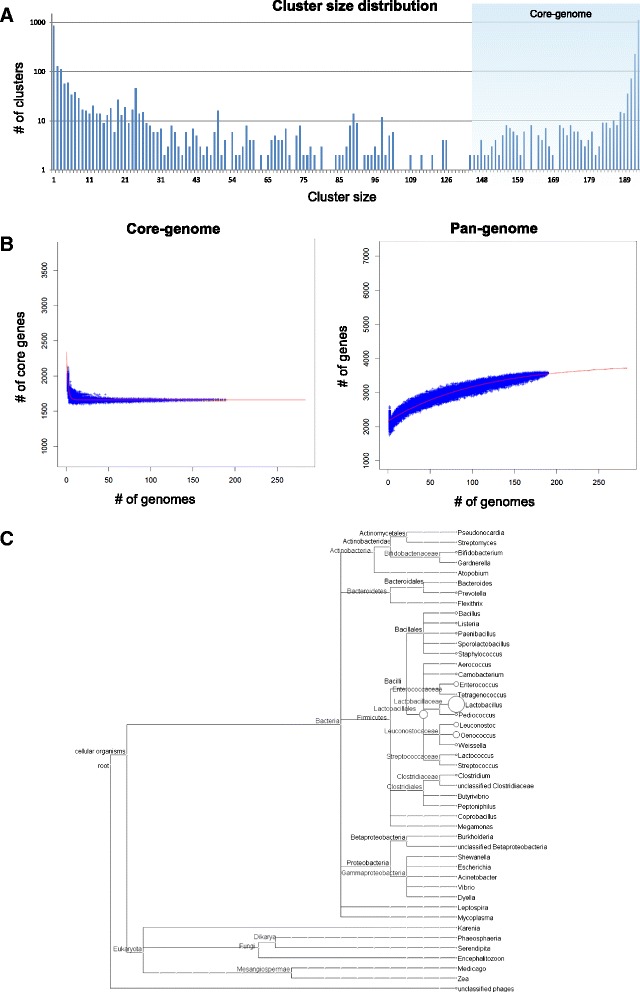
The core- and pan-genomes of *O. oeni*. **a** Distribution of protein cluster sizes generated from the comparison of 191 genomes. **b** Calculation of core- and pan-genome sizes including exponential law models to fit the medians. **c** Distribution of BLAST best-hits by genus for clusters with no *O. oeni* match in the NCBI non-redundant dataset. The size of the circles represents the number of assigned hits

**Fig. 3:**
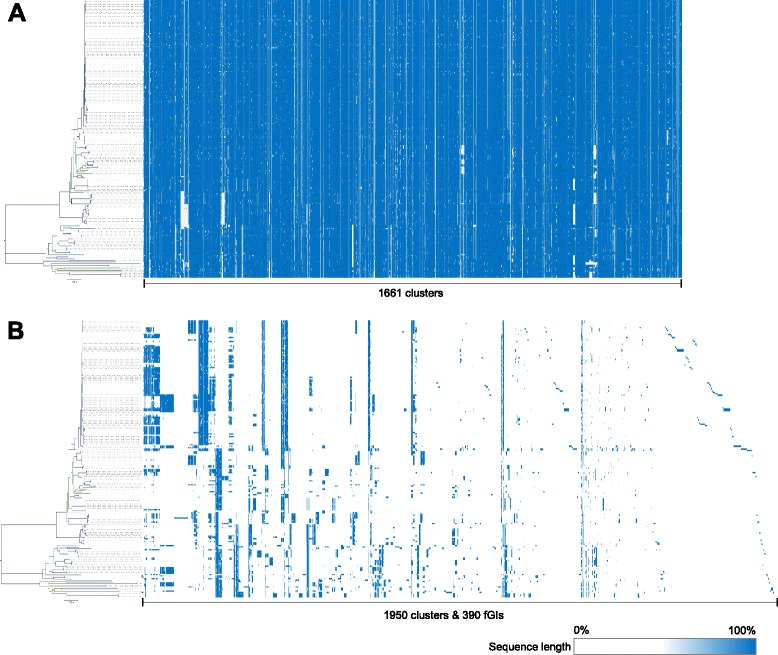
Visualisation of the core-genome and fGI assemblies. Full versions of the annotated assemblies are available in Additional file 3. **a** Core-genome assembly of 1661 clusters. **b** Concatenated fGI assemblies of 1950 clusters into 390 fGIs

**Fig. 4:**
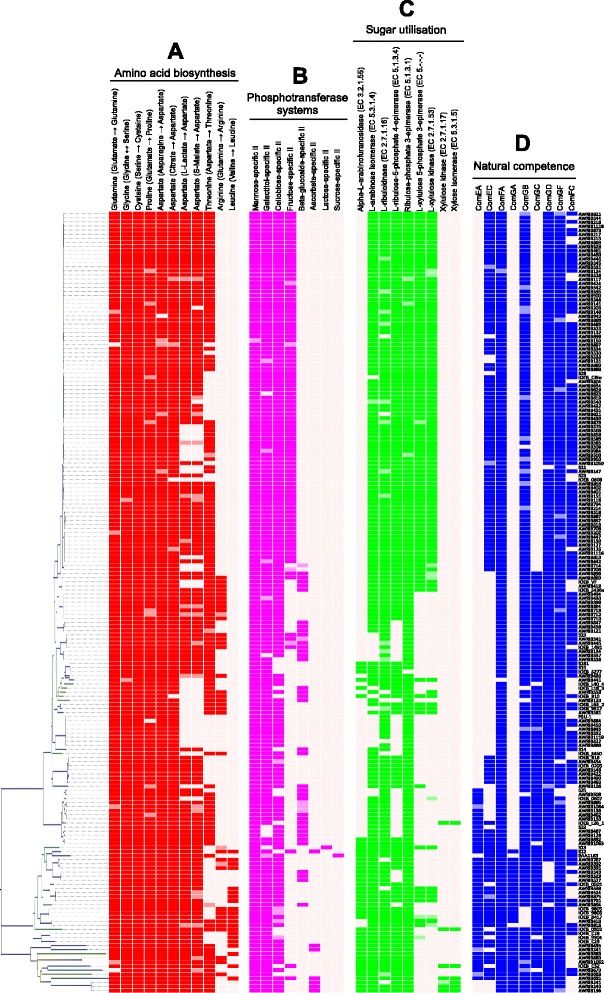
Intra-specific differences in amino acid biosynthesis, sugar transport and utilisation and natural competence. ORFs which contained a contig break are shaded in a lighter colour. **a** Intra-specific differences in amino acid biosynthesis. Each pathway requires multiple enzymes, as described by their KEGG module numbers. **b** Intra-specific differences in PTS components. Each sugar-specific system requires multiple subunits (typically IIA, IIB, IIC and occasionally IID). **c** Intra-specific differences in the genes involved in five-carbon sugar utilisation, as described in Fig. 6. **d** Intra-specific differences in the genes encoding natural competence proteins

**Fig. 5:**
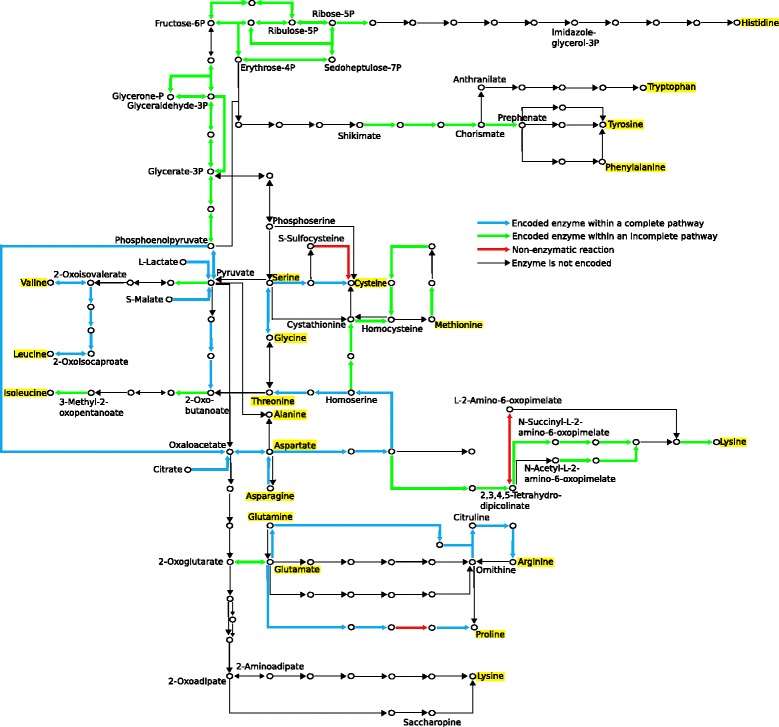
Overview of amino acid biosynthesis pathways in *O. oeni.* KEGG, RAST and BLAST annotations were used determine the presence of ORFs associated with amino acid biosynthesis across 191 strains. Pathways containing the full set of required genes, mostly between two amino acids (highlighted in yellow), are highlighted in blue and represented in Fig. 4a. ORFs forming incomplete pathways are highlighted in green. Pathways to make nine different amino acids were observed

**Fig. 6:**
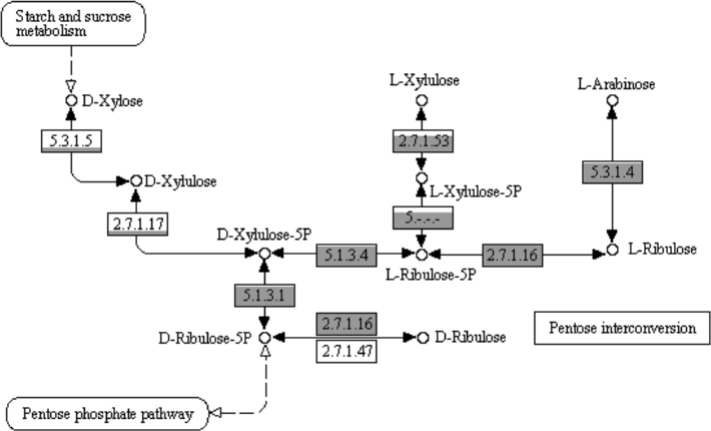
Variations in five-carbon sugar utilisation in *O. oeni*. The predicted pathways for the assimilation of xylose, arabinose and xylulose and the individual enzymatic steps with their corresponding EC numbers are indicated. The grey shading represents the number of strains out of 191 which contain that enzyme. The boxes are shaded relative to each other on a square-root scale. EC 2.7.1.16 was most common, present in 176 strains

**Fig. 7:**
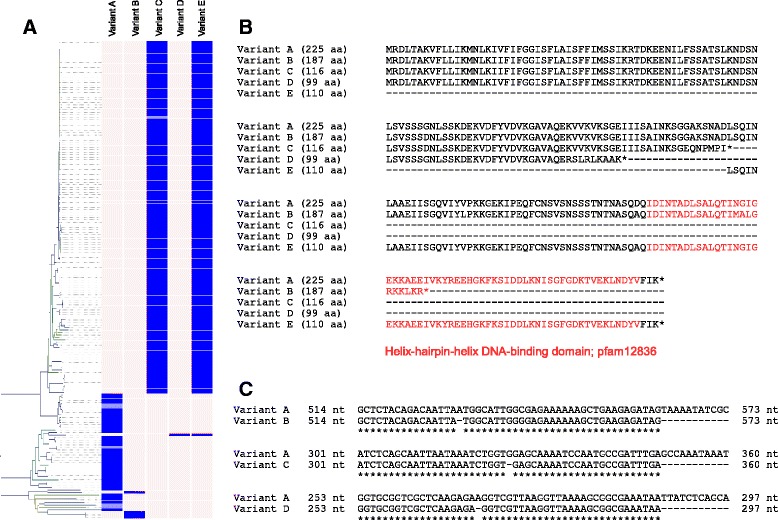
Intra-specific variation in the gene encoding the ComEA transmembrane DNA receptor. **a** Five variants were found to map to specific branches of the genetic relatedness dendrogram. Strains which contained a contig break in the ORF encoding ComEA are shaded in light blue and assigned to the likely variant category. **b** Alignment of predicted ComEA peptide sequences showing full-length (Variant A) and truncated (Variants B to E) versions. Variants B, C and D contained frameshift mutations resulting in prematurely-encoded stop codons which resulted in an additional ORF being predicted *in silico* (Variant E). The C-terminal DNA-binding motif is highlighted in red and is not encoded by Variants C and D. Variant B contains a premature stop within the DNA-binding domain and still corresponds with genetically-distant strains. Variant D represents a frameshift mutation unique to the BAA-1163 strain. **c** Nucleotide sequence alignment highlighting single nucleotide deletions causing frameshift mutations and truncation of the ComEA peptide sequence

## References

[1] Sternes PR, Borneman AR (2016). Consensus pan-genome assembly of the specialised wine bacterium *Oenococcus oeni*. BMC Genomics.

