# Supplementary figures and images for: Consensus pan-genome assembly of the specialised wine bacterium Oenococcus oeni
Source: BMC Genomics. 2016 Apr 27;17:308. doi: 10.1186/s12864-016-2604-7 (PMC4847254; doi:10.1186/s12864-016-2604-7)

**Core-genome**

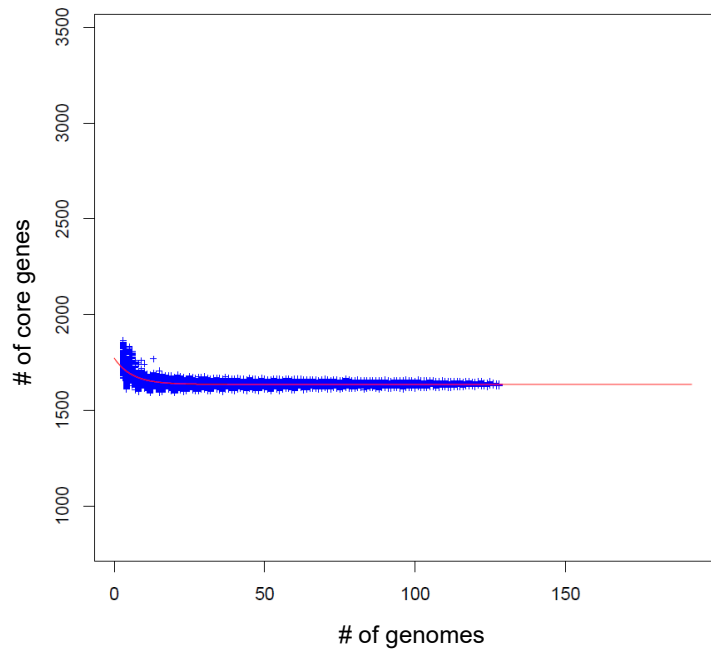

**Pan-genome**

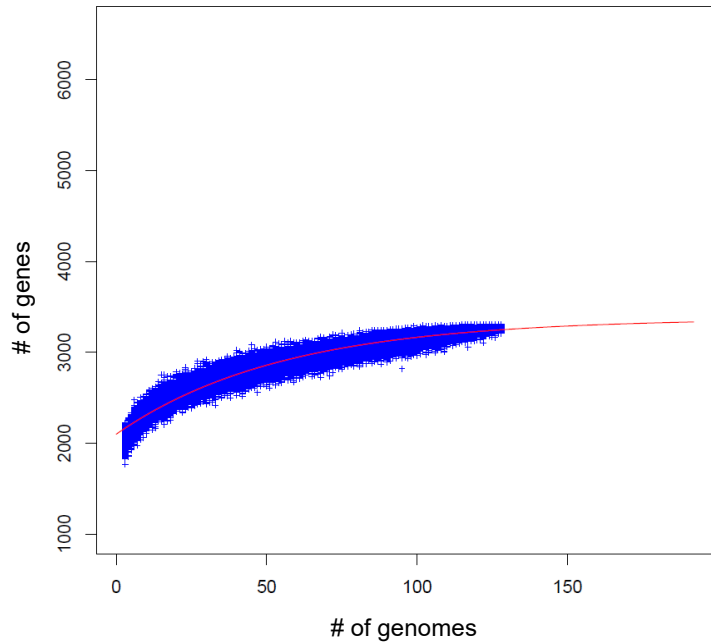

Supplement: Additional file 1: Figure S1. — Calculation of core- and pan-genome sizes including exponential law models to fit the medians. These calculations check for bias when a high number of closely-related strains are included in the core- and pan-genome size calculations. 60 closely-related genomes from Group A in Fig. 1 were excluded from the calculation to check for bias in Fig. 2b. (PDF 70 kb) [file 12864_2016_2604_MOESM1_ESM.pdf]

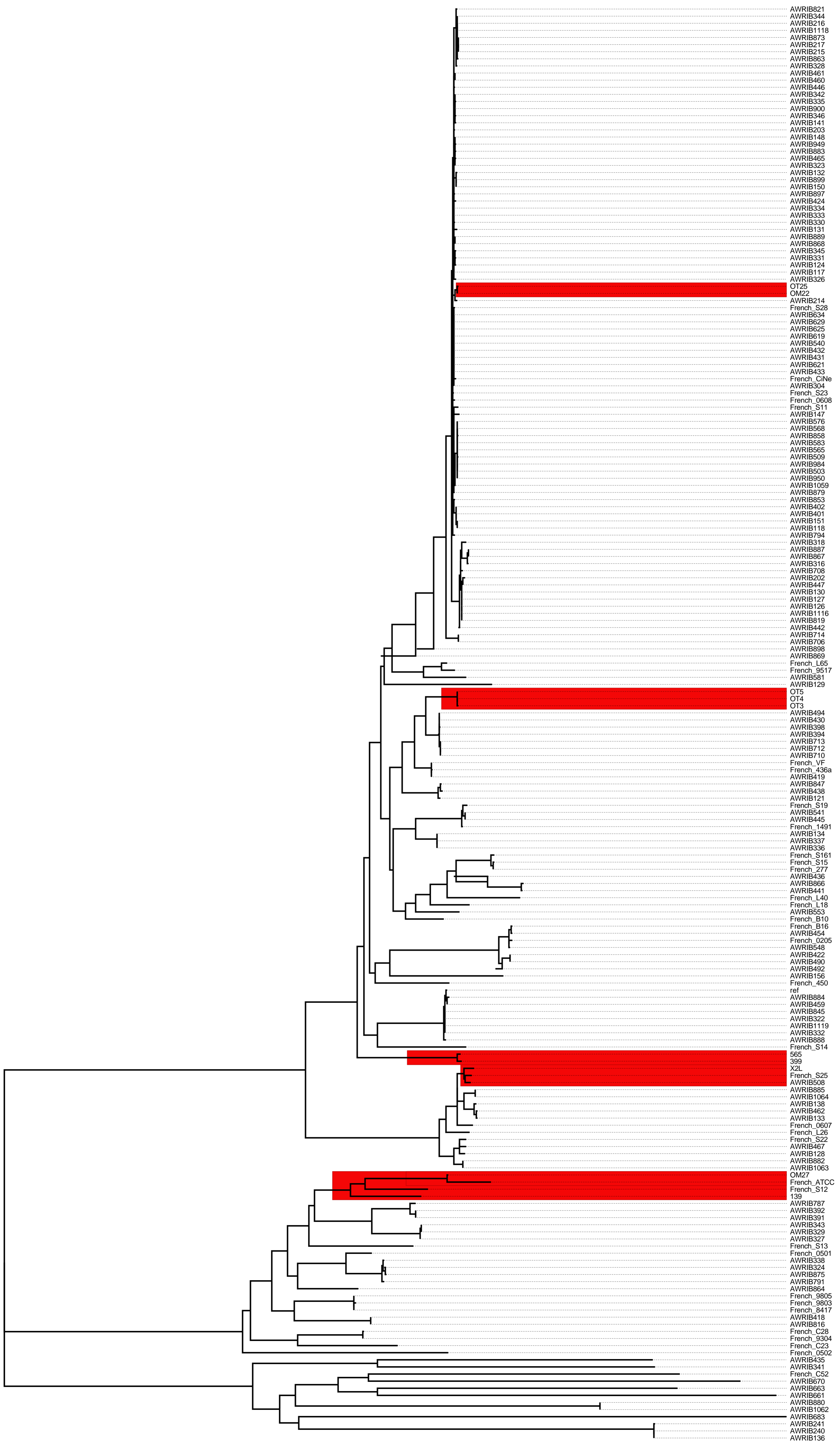

7.0E-4

Supplement: Additional file 2: Figure S2. — Updated neighbour-joining phylogeny to include recently released Italian and South American O. oeni strains. Neighbour-joining phylogeny based on whole-genome alignments of 191 O. oeni strains used for the pan-genome construction in addition to 10 strains from Italy (OM27, OM22, OT25, OT3, OT4, OT5), Argentina (XL2) and Chile (139, 399, 565) for which whole-genome data is now available. Phylogenomic clades containing the additional strains are highlighted in red. (PDF 10 kb) [file 12864_2016_2604_MOESM2_ESM.pdf]
