# Supplementary material for: Consensus pan-genome assembly of the specialised wine bacterium Oenococcus oeni
Source: BMC Genomics. 2016 Apr 27;17:308. doi: 10.1186/s12864-016-2604-7 (PMC4847254; doi:10.1186/s12864-016-2604-7)

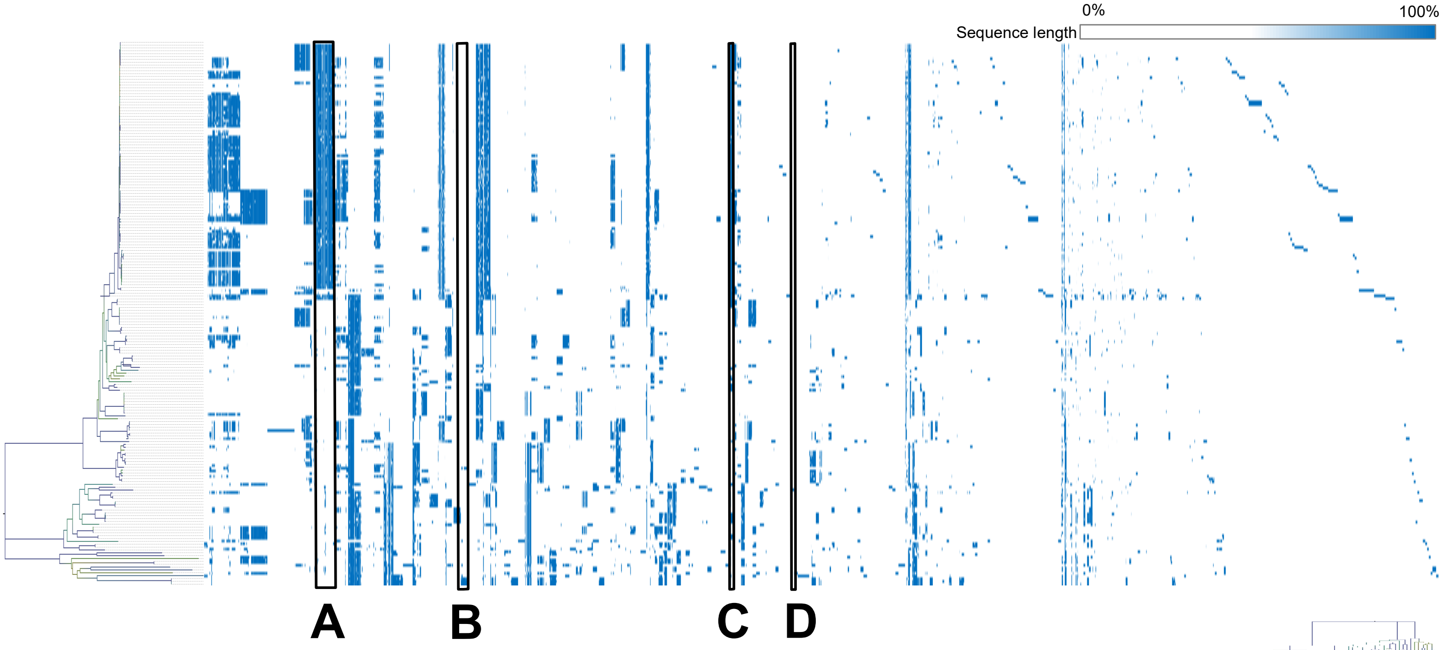

A

Position	fGI #	Cluster #	RAST annotation	BLAST top hit
258	12	CL_1839_	Tyrosine-protein kinase transmembrane modulator EpsC	capsular polysaccharide biosynthesis protein [Oenococcus oeni]
259	12	CL_1838_	capsular polysaccharide biosynthesis protein	hypothetical protein [Oenococcus oeni]
260	12	CL_1837_	Manganese-dependent protein-tyrosine phosphatase (EC 3.1.3.48)	hypothetical protein [Oenococcus oeni]
261	12	CL_1836_	Undecaprenyl-phosphate galactosephosphotransferase (EC 2.7.8.6)	sugar transferase [Oenococcus oeni]
262	12	CL_1835_	Polysaccharide biosynthesis protein CpsF	UDP-N-acetylglucosamine:LPS N-acetylglucosamine transferase [Oenococcus oeni]
263	12	CL_1834_	Glycosyl transferase CpsG	multidrug MFS transporter [Oenococcus oeni]
264	12	CL_1833_	-	hypothetical protein AWRIB576_968 [Oenococcus oeni AWRIB576]
265	12	CL_1832_	-	glycosyltransferase [Oenococcus oeni]
266	12	CL_1831_	-	glycosyltransferase family 2 [Oenococcus oeni]
267	12	CL_1830_	-	glycosyltransferase family 2 [Oenococcus oeni]
268	12	CL_1829_	-	hypothetical protein [Oenococcus oeni]
269	12	CL_1828_	Holliday junction DNA helicase RuvA	glycosyltransferase family 1 [Oenococcus oeni]
270	12	CL_1827_	-	hypothetical protein [Oenococcus oeni]
271	12	CL_1826_	-	hypothetical protein [Oenococcus oeni]
272	12	CL_1825_	-	hypothetical protein [Oenococcus oeni]
273	12	CL_1824_	-	hypothetical protein [Oenococcus oeni]
274	12	CL_1823_	-	hypothetical protein [Oenococcus oeni]
275	12	CL_1822_	-	transcriptional antiterminator [Oenococcus oeni]
276	12	CL_1821_	6-phospho-beta-glucosidase (EC 3.2.1.86)	beta-glucosidase [Oenococcus oeni]
277	12	CL_1820_	Transcription antiterminator, BglG family	transcriptional antiterminator [Oenococcus oeni]
278	12	CL_1819_	-	PTS mannose transporter subunit IIAB, partial [Oenococcus oeni]
279	12	CL_1818_	PTS system, fructose-specific IIB component (EC 2.7.1.69)	PTS fructose transporter subunit IIB [Oenococcus oeni]
280	12	CL_1817_	PTS system, IIC component	hypothetical protein [Oenococcus oeni]
281	12	CL_1816_	PTS system, IIC component	PTS mannose transporter subunit IIAB [Oenococcus oeni]
282	12	CL_1815_	Alpha-mannosidase (EC 3.2.1.24)	hypothetical protein [Oenococcus oeni]
283	12	CL_2042_	-	hypothetical protein [Oenococcus oeni]
284	12	CL_2041_	Oligopeptide ABC transporter, periplasmic oligopeptide-binding protein OppA (TC 3.A.1.5.1)	peptide ABC transporter substrate-binding protein [Oenococcus oeni]
285	12	CL_2040_	Deblocking aminopeptidase (EC 3.4.11.-)	peptidase M42 [Oenococcus oeni]
286	12	CL_2039_	FIG00628259: hypothetical protein	peptidase [Oenococcus oeni]
287	12	CL_2038_	Substrate-specific component YkoE of thiamin-regulated ECF transporter for HydroxyMethylPyrimidine	ABC transporter permease [Oenococcus oeni]

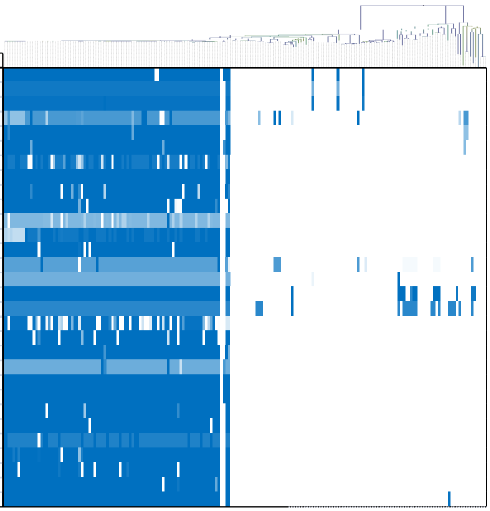

B

Position	fGI #	Cluster #	RAST annotation	BLAST top hit
609	28	CL_17065_	-	hypothetical protein [Oenococcus oeni]
610	28	CL_17066_	Xylulose kinase (EC 2.7.1.17)	xylulose kinase [Oenococcus oeni]
611	28	CL_17067_	Xylose isomerase (EC 5.3.1.5)	xylose isomerase [Lactobacillus casei]
612	28	CL_19301_	Xylose repressor	transcriptional regulator [Lactobacillus buchneri]
613	28	CL_17068_	D-xylose proton-symporter XylT	hypothetical protein [Oenococcus oeni]
614	28	CL_17069_	-	hypothetical protein [Oenococcus oeni]
615	28	CL_17070_	PTS system, mannose-specific IIB component (EC 2.7.1.69)	PTS sorbose transporter subunit IIB [Oenococcus oeni]
616	28	CL_17071_	Alpha-xylosidase (EC 3.2.1.-)	family 31 glucosidase [Oenococcus oeni]
617	28	CL_17072_	PTS system, mannose-specific IIC component	PTS mannnose transporter subunit IIC [Oenococcus oeni]
618	28	CL_17073_	PTS system, mannose-specific IID component	PTS mannose transporter subunit IID [Oenococcus oeni]
619	28	CL_17074_	Trehalose-6-phosphate hydrolase (EC 3.2.1.93)	oligo-1,6-glucosidase [Oenococcus oeni]
620	28	CL_19206_	Ferrochelatase, protoheme ferro-lyase (EC 4.99.1.1)	ferrochelatase [Enterococcus moraviensis]

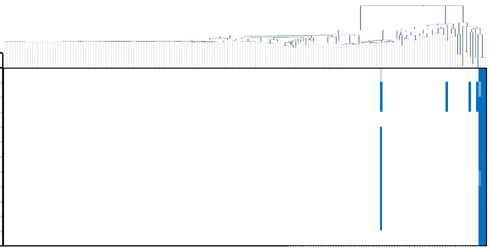

C

Position	fGI #	Cluster #	RAST annotation	BLAST top hit
1525	92	CL_2097_	-	transcriptional regulator [Oenococcus oeni]
1526	92	CL_2098_	Glutathione S-transferase (EC 2.5.1.18)	glutathione S-transferase [Oenococcus oeni]
1527	92	CL_2099_	L-ribulose-5-phosphate 4-epimerase (EC 5.1.3.4)	L-ribulose-5-phosphate 4-epimerase [Oenococcus oeni]
1528	92	CL_2100_	L-xylulose 5-phosphate 3-epimerase (EC 5.1.3.-)	L-xylulose 5-phosphate 3-epimerase [Oenococcus oeni]
1529	92	CL_2101_	L-xylulokinase (EC 2.7.1.53)	carbohydrate kinase [Oenococcus oeni]
1530	92	CL_2102_	-	hypothetical protein [Oenococcus oeni]

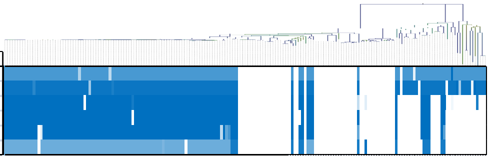

D

Position	fGI #	Cluster #	RAST annotation	BLAST top hit
1718	111	CL_17654_	-	PTS system, glucose/sucrose specific IIA subunit [Oenococcus oeni]
1719	111	CL_17655_	-	sucrose-6-phosphate hydrolase, partial [Lactobacillus vini]
1720	111	CL_17656_	Sucrose-6-phosphate hydrolase (EC 3.2.1.B3)	sucrose-6-phosphate hydrolase [Lactobacillus mali]
1721	111	CL_17657_	PTS system, sucrose-specific IIBC component (EC 2.7.1.69)	PTS sugar transporter [Oenococcus oeni]
1722	111	CL_17658_	-	sucrose operon repressor [Lactobacillus mali]
1723	111	CL_17659_	-	sucrose operon repressor [Lactobacillus mali]

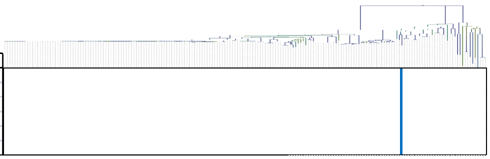

Supplement: Additional file 4: Figure S3. — fGIs conferring intra-specific differences in PTS enzymes and sugar utilisation. Graphical representation of four annotated fGIs and their phylogenomic relationship. A. A large fGI containing 29 genes, two of which encode fructose-specific IIB and IIC PTS components, completing the full suite of fructose-specific II components (IIA, IIB and IIC) in those strains. B. An fGI containing two enzymes, xylulose kinase EC 2.7.1.17 and xylose isomerise EC 5.3.1.5, and potentially related genes which is predicted to confer the ability to interconvert xylose to xylulose-5P. C. An fGI containing three enzymes, L-ribulose-5-phosphate 4-epimerase EC 5.1.3.4, L-xylulose 5-phosphate 3-epimerase EC 5.-,-,- and L-xylulokinase EC 2.7.1.53, and potentially related genes which is predicted to confer the ability to interconvert L-xylulose to D-xylulose-5P. D. A strain-specific fGI containing sucrose-related genes which encode sucrose-specific IIA and IIBC PTS subunits, sucrose operon repressors and partial and complete sucrose-6-phosphate hydrolases, potentially conferring the ability to perceive, transport and utilise sucrose. (PDF 1476 kb) [file 12864_2016_2604_MOESM4_ESM.pdf]
